# *ScDREB10*, an A-5c type of *DREB* Gene of the Desert Moss *Syntrichia caninervis*, Confers Osmotic and Salt Tolerances to *Arabidopsis*

**DOI:** 10.3390/genes10020146

**Published:** 2019-02-14

**Authors:** Xiaoshuang Li, Yuqing Liang, Bei Gao, Meiheriguli Mijiti, Tohir A. Bozorov, Honglan Yang, Daoyuan Zhang, Andrew J. Wood

**Affiliations:** 1 CAS Key Laboratory of Biogeography and Bioresource in Arid Land, Xinjiang Institute of Ecology and Geography, Chinese Academy of Sciences, Xinjiang Urumqi 830011, China; lixs@ms.xjb.ac.cn (X.L.); liangyuqing14@mails.ucas.ac.cn (Y.L.); mika1237@163.com (M.M.); tohirbozorov@yahoo.com (T.A.B.); yanghonglan@ms.xjb.ac.cn (H.Y.); 2 University of Chinese Academy of Sciences, Beijing 100049, China; 3 School of Life Sciences and State Key Laboratory of Agrobiotechnology, The Chinese University of Hong Kong, Hong Kong 852, China; gaobei@link.cuhk.edu.hk; 4 Institute of Genetics and Plants Experimental Biology, Uzbek Academy of Sciences, Yukori-Yuz 111226, Kibray, Tashkent Region, Uzbekistan; 5 Department of Plant Biology, Southern Illinois University, Carbondale, IL 62901-6899, USA; wood@plant.siu.edu

**Keywords:** *Syntrichia caninervis*, DREB transcription factor, drought stress, salt stress, ROS-scavenging ability

## Abstract

Drought and salinity are major factors limiting crop productivity worldwide. DREB (dehydration-responsive element-binding) transcription factors play important roles in plant stress response and have been identified in a wide variety of plants. Studies on DREB are focused on the A-1 (*DREB1*) and A-2 (*DREB2*) groups. Studies on A-5 group DREBs, which represent a large proportion of the DREB subfamily, is limited. In this study, we characterized and analyzed the stress tolerance function of *ScDREB10*, an A-5c type *DREB* gene from the desert moss *Syntrichia caninervis*. Transactivation assay in yeast showed that ScDREB10 had transactivation activity. Transient expression assay revealed that ScDREB10 was distributed both in the nucleus and cytosol of tobacco leaf epidermal cells. Overexpression of *ScDREB10* significantly increased the germination percentage of *Arabidopsis* seeds under osmotic and salt stresses, and improved the osmotic and salt stress tolerances of *Arabidopsis* at the seedling stage and is associated with the expression of downstream stress-related genes and improved reactive oxygen species (ROS) scavenging ability. Our study provides insight into the molecular mechanism of stress tolerance of A-5 type DREB proteins, as well as providing a promising candidate gene for crop salt and drought stress breeding.

## 1. Introduction

Drought and salinity are common abiotic stress factors that seriously affect plant growth and development [1,2]. Drought stress can lead to osmotic stress and oxidative damage, while salt stress primarily imposes osmotic stress and ion toxicity [3]. These stresses can disrupt cellular structure and impair key physiological functions, and in extreme cases, cause plant death [4]. To cope with these abiotic stresses, plants have evolved a series of response including physiological, metabolic and molecular processes [4,5].

DREB (dehydration-responsive element-binding) proteins belong to the AP2/ERF (APETALA2/ethylene-responsive Element Binding Factor) gene family, which comprises one of the largest group of plant transcription factors and is involved in various plant stress responses [6,7,8,9,10]. The *DREB* genes were originally isolated from *Arabidopsis thaliana* [11], and in the past two decades of research, *DREBs* have been widely identified in various plants, such as rice, soybean, maize ([7,8,9], as well as some desert plants, such as *Eremosparton songoricum*, *Caragana korshinskiis*, and *Tamarix hispida* [12,13,14], and they have proved to play central roles in plant abiotic stress responses [15,16]. DREB proteins were divided into six groups termed A-1 to A-6 based on the similarities of the AP2 domain [17]. DREB proteins showed functional divergence in stress responses, for example, A-1 type of DREBs primarily respond to cold stress [11], A-2 type DREBs respond to drought, salt, and heat stresses [9,17], and A3 type of DREBs function in regulating seed responses to ABA (abscisic acid) [18]. Among them, A-1 (*DREB1*) and A-2 (*DREB2*) were extensively studied and are considered to be the two main groups of DREBs primarily involved in the regulation of plant abiotic stress response [9,10,11,15,16,17]. A-5 DREBs contains a large number of gene members while are rarely studied. Li et al. reported that A-5 type of *DREB* genes also plays important roles in plant stress responses [19]. Therefore, more members of the A-5 DREB protein should be studied to better understand their functions and regulation mechanisms in plant tolerance to stress.

*Syntrichia caninervis*, a widespread moss species in biological soil crusts of the Gurbantunggut Desert of Northwestern China [20], is considered as an excellent model for understanding plant desiccation tolerance mechanism and a good plant source for identification of stress-related genes [19,21,22,23,24,25]. In a previous study, we generated a dehydration–rehydration transcriptome profile of *S. caninervis* [24] and selected ten *DREB* genes which belonged to the A-5 group of DREB for analysis of their abiotic stress response and stress tolerance [25]. Among them, *ScDREB10* (Genbank number: KU613418) had the longest ORF (open reading frame) with 1165 bp encoding 371 amino acids [25]. Stress tolerance ability evaluation in transgenic yeast system demonstrated that *ScDREB10* is a promising stress tolerant gene which conferred multiple stress tolerances in yeast cells, especially for osmotic and salt stresses [25]. In the present study, we further evaluated the stress tolerance function of the *ScDREB10* gene in *Arabidopsis* at phenotypic, physiological, and molecular levels. Our results showed that *ScDREB10*, an A-5c type of *DREB* gene, located in both the nucleus and cytoplasm, had transactivation activity, and the overexpressing-*ScDREB10* had significantly enhanced osmotic and salt stress tolerances in *Arabidopsis* at germination and young seedling stages. ScDREB10 transgenic *Arabidopsis* accumulated less reactive oxygen species (ROS) and had higher antioxidant enzyme activities to scavenge ROS and highly induced the gene expression of a series of downstream stress-related genes. *ScDREB10* may be an excellent candidate gene for improving crop drought and salt stress tolerance. 

## 2. Materials and Methods

### 2.1. Plant Materials and Growth Conditions

The *Arabidopsis thaliana* L. Col-0 ecotype was used as the genetic background/wild-type (WT) for the transgenic plants generated in this study. Plants were grown in a greenhouse under standard growth conditions (22 ± 2 °C: 16 h light/8 h dark photoperiod and 60 to 70% relative humidity [26].

### 2.2. DNA/Protein Sequence and Phylogenetic Analyses

The predicted protein sequences of classic DREBs which represented the A1-A6 groups from the model plant *A. thaliana*, rice, and other plants with high sequence similarities with ScDREB10 were retrieved from the NCBI (National Center for Biotechnology Information) Entrez database (http://www.ncbi.nlm.nih.gov). Multiple sequence alignments of amino acid sequences from different species were performed by ClustalW, and phylogenetic trees were constructed using the neighbor-joining (NJ) method and a bootstrap test with 1000 replicates by MEGA 6.06 software (Poisson correction and pairwise deletion) [27,28]. 

### 2.3. Subcellular Localization Analysis

The ORF of ScDREB10 without a stop codon was amplified from pMD18-T-*ScDREB10* positive plasmids using gene-specific primers containing an *Sma* I restriction site, and the PCR product was fused to the N-terminus of the green fluorescent protein (GFP) gene driven by the CaMV 35S promoter in pBI121 vector using an in-fusion PCR cloning system (Clontech, Mountain View, CA, USA). The primers for this experiment are listed in Table S1. Both the transient expression of the *35S*:*ScDREB10-GFP* fusion gene and the *35S*:*GFP* control plasmids were introduced into four-week-old wild-type (WT) tobacco (*Nicotiana bentamiana*) leaf epidermal cells via *Agrobacterium*-mediated leaf infiltration for transient transformation [29]. Transformed cells expressing ScDREB10-GFP were observed two days after *Agrobacterium*-infiltration using confocal laser scanning microscopy LSM800 (Zeiss, Jena, Germany). GFP fluorescence was imaged in a single channel setting with 488 nm for GFP excitation.

### 2.4. Analysis of Transactivation Activity of ScDREB10

To investigate the transcriptional activity of ScDREB10, the yeast two-hybrid system (Y2H) with pGBKT7 vector and Y2H yeast strain were used (Clontech). The coding sequence of ScDERB10 was fused in frame with the GAL4 DNA-binding domain (pGBKT7 vector) to produce the fusion construct of BD-ScDREB10 using the in-fusion PCR cloning system (Clontech); the full-length ORF of ScDREB10 was amplified from pMD18-T plasmids by PCR using gene-specific primers containing *Eco*R I and *Bam*H I restriction sites, and the PCR product was inserted into the *Eco*R I/*Bam*H I pGBKT7 vector. The construct was subsequently introduced into yeast Y2H Gold cells (Clontech). The yeast positive transformants were adjusted to an OD600 of 2.0, and the yeast cells were then 10-fold serially diluted and dropped with 2 µL on synthetic dropout (SD) medium without tryptophan (SD/−Trp), without tryptophan and histidine (SD/−Trp−His), and with SD/−Trp−His plates containing x-α-gal (5-Bromo-4-chloro-3-indolyl α-d-galactopyranoside) (SD/−Trp−His + x-α-gal). Yeast cells expressing containing the pGBKT7 empty vector or expressing GAL4 were used as the negative and positive control, respectively. The plates were incubated at 30 °C for 2 to 4 days before photographing. The primer information was listed in Table S1.

### 2.5. Generation of ScDREB10-Overexpressing Arabidopsis

The restriction enzymes *Kpn*I and *Xba*I were used to digest both the pYES2-*ScDREB10* positive plasmid [25] and the plant expression vector pCAMBIA1301 containing the CaMV 35S promoter. The *ScDREB10* ORF was ligated into the digested pCAMBIA1301 vector and introduced into wild-type (WT) *Arabidopsis* plants by the floral dip method [30]. Seeds of T1 to T3 generation of transgenic plants were selected on Murashige and Skoog (MS) medium containing 80 mg L^−1^ hygromycin. The hygromycin-resistant T1 seedlings were tested by PCR analysis and sequencing, the T3 homozygous lines produced from the T1 plants expressing *ScDREB10* genes were collected for further functional analysis of stress tolerance. 

### 2.6. Evaluation of the Osmotic and Salt Stress Tolerances of Transgenic Arabidopsis at Germination Stage

For germination, both the WT and transgenic T3 seeds were surface-sterilized and placed on MS agar plates (control) or MS agar plates supplied with either 250 mM mannitol (osmotic stress) or 150 mM NaCl (salt stress). The plates were placed on greenhouse benches at 22 ± 2 °C with 16 h light/8 h dark photoperiod and 60 to 70% relative humidity. Germination percentage was calculated after 7 days (the germination percentage was calculated by the number of germinated seeds divided by the total number of seeds, represented as a percentage) plants were photographed before and after stress treatments. The germination percentage statistics were calculated by at least 50 seeds for each line with three biological replicates for each treatment.

### 2.7. Evaluation of Osmotic and Salt Stress Tolerances of Transgenic Arabidopsis at Seedling Stage

For the seedlings stage, one-week-old seedlings of WT and transgenic *Arabidopsis* cultured on MS medium were transferred on the MS agar plates (control), MS agar plates supplied with 250 mM mannitol (osmotic stress) or 150 mM NaCl (salt stress), and maintained for 7 days. Our preliminary data suggested these two treatments are sufficient to trigger stress and that 7 days was when a visible phenotype was evident. More than 20 seedlings of WT and each transgenic line were used in this experiment for further calculating the root length, fresh weight, and lateral root number. Photographs for phenotype observation were taken before and after stress treatments. After stress treatments, some of these seedlings were collected and used for the diaminobenzidine (DAB) and nitrotetrazolium blue chloride (NBT) staining, and some seedlings were harvested by immediate flash freezing in liquid nitrogen for the physiological indexes’ measurement. WT and transgenic plants (100 mg fresh weight) were ground with ice-cold 0.1 mol L^−1^ potassium phosphate buffer (pH 7.4; 1:9 *w*/*v*), homogenates were clarified by centrifugation at 8000× *g* for 10 min at 4 °C. The supernatants were prepared for the measurement of hydrogen peroxide (H_2_O_2_) levels and malondialdehyde (MDA) content, superoxide dismutase (SOD, EC 1.15.1.1) and peroxidase (POD, EC 1.11.1.7) activities using assay kits according to the manufacturer’s instructions (kit no. A064, A003-3, A001-1, A084-3; Nanjing Jiancheng Bioengineering Institute, China) as Shi et al. [31,32] described.

### 2.8. Determination of the Potential Down-Stream Genes of ScDREB10

Seven-day-old seedlings of WT and transgenic plants grown on MS medium were transferred onto MS medium supplied with 250 mM mannitol (osmotic stress) or 150 mM NaCl (salt stress) for 7 days, and seedlings were subsequently harvested for RNA extraction using the Plant RNA Kit (OMEGA, Guangzhou, China). First-strand cDNA was synthesized using 1 μg RNA with PrimeScript RT reagent Kit (TaKaRa, Tokyo, Japan). Quantitative real-time PCR (RT-qPCR) was performed on CFX-96 Real-Time System (Bio-Rad, Hercules, CA with SYBR Premix Ex Taq™ II (TaKaRa). The *α-TUBULIN* (At1g50010) and *UBIQUTIN10* (At4g05320) genes of *Arabidopsis* were used as internal controls for RT-qPCR normalization [33,34]. The PCR condition was as follows: initial denaturation step of 30 s at 95 °C, 40 cycles of PCR (95 °C for 5 s, 58–60 °C for 30 s). Three biological and technological replicates for each sample were performed. Relative expression of the detected genes was calculated using the 2^−ΔΔ*C*t^ method [35]. The primers used for RT-qPCR analysis were listed in Table S2.

### 2.9. Statistical Analysis

All data in this study are expressed as the mean values ± SE with at least three replicates. The data were analyzed by LSD multiple comparison tests in the one-way analysis of variance (ANOVA) program of SPSS 19.0 software (Windows, SPSS Inc., Chicago, IL, USA), and differences were considered statistically significant at * *P* < 0.05 and very significant at ** *P* < 0.01. All figures were generated using Sigmaplot 12.0 (Systat Software, San Jose, CA, USA) and Adobe Illustrator CS software.

## 3. Results

### 3.1. Phylogenetic Analysis of ScDREB10

**Figure 1:**
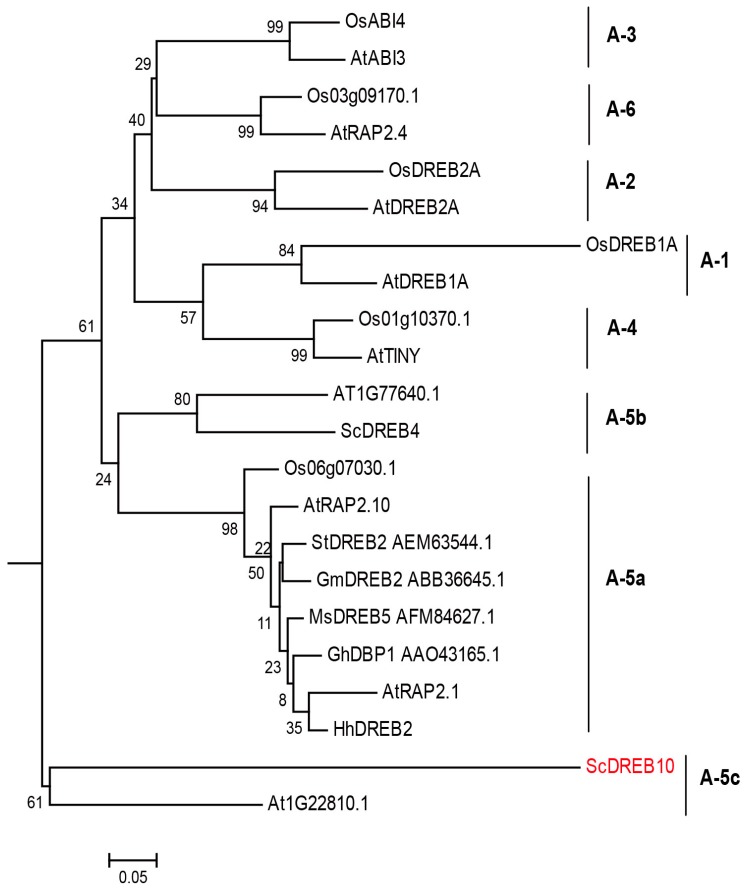
Phylogenetic analyses of APETALA2 (AP2) domains of ScDREB10 and other classic plant dehydration-responsive element-binding (DREBs). Phylogenetic tree of ScDREB10 with other 21 DREBs from different plant species representing A1-A6 groups was constructed using the neighbor-joining method, the evolutionary distances were computed using the Poisson correction method with pairwise deletion. Bootstrap values from 1000 replicates were used to assess the robustness of the tree. All amino acid sequences were retrieved from GenBank: ScDREB4 (KU613412) and ScDREB10 (KU613418) are from *Syntrichia caninervis*; eight DREBs are from *Arabidopsis thaliana* including AtDREB2A (BAA33794), AtDREB1A (BAA33791), AtABI3 (AT2G40220.1), AtRAP2.1 (OAP00017), TINY (AT5G25810.1), ATRAP2.10 (AT4G36900), AT1G22810.1, AT1G77640.1; six DREB*s* are from *Oryza sativa*, OsDREB2A (AFB77198), OsDREB1A (AEW67332), OsABI4 (Os05g28350.1), Os03g09170.1, Os01g10370.1, Os06g07030.1; StDREB2 (AEM63544.1) is from potato; GhDBP1 (AAO43165.1) is from cotton; GmDREB2 (ABB36645.1) is from soybean; MsDREB5 (AFM84627.1) is from *Malus sieversii*; HhDREB2 (ACJ66376.1) is from *Halimodendron halodendronand*.

*ScDREB10* previously was classified to the A-5 type of DREBs, is 1165 bp in length with an ORF encoding a predicted polypeptide of 371 amino acids (Genbank number: KU613418 [25]. The A-5 DREB group can be divided into three subgroups: A-5a, A-5b, and A-5c based upon motif composition and sequence similarities. The AP2 domains of ScDREB10 with 21 other classic DREBs from different plant species representative of A1-A6 groups were used to construct a phylogenetic tree to further confirm the sequence classification. The gene tree showed that ScDREB10 grouped as a separate A-5c clade with AT1G22810 (Figure 1), although the vast majority of identified A-5 proteins belonged to A-5a subgroup, such as RAP2.1 (related to AP2 1) and GhDBP1 (*Gossypium hirsutum* DRE-binding protein 1). Furthermore, pairwise distance analysis showed that the lowest genetic distance is 0.788 with OsDREB2A, whereas the highest genetic distance is 1.033 with OsDREB1A (Table S3). The pairwise sequence similarities of AP2 domain of 22 DREBs showed that ScDREB10 had very low sequence similarities with other DREBs, and the sequence identities of AP2 domain is below 0.5 even compared within A-5 type group (Table S3).

### 3.2. Localization and Transactivation Activity Analysis of the ScDREB10 Protein

**Figure 2:**
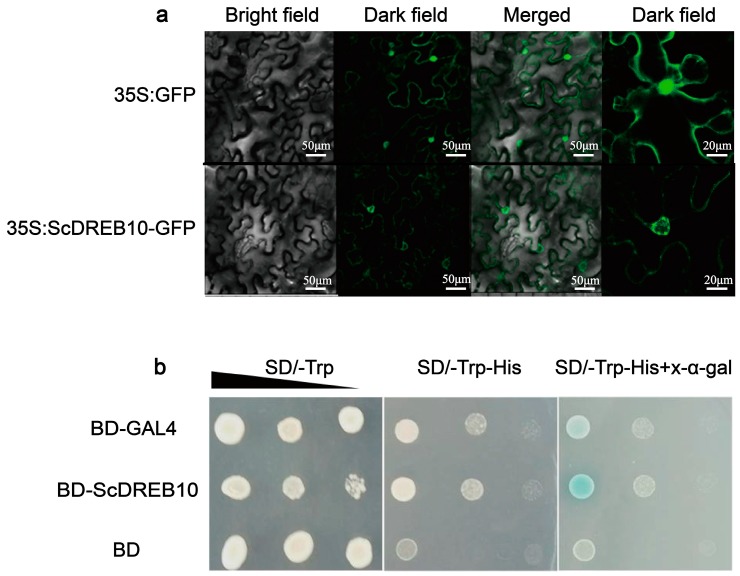
Analysis of subcellular localization and trans-activation activity of ScDREB10. (**a**) Subcellular localization of ScDREB10 in tobacco leaf epidermal cells. 35S:ScDREB10-GFP fusion protein and 35S:GFP control were transiently expressed in tobacco (*Nicotiana bentamiana*.) leaf epidermal cells and transformed cells were observed with a laser scanning confocal microscope after incubation for 24 h in the dark. The images were presented bright field, dark field, and merge of bright field and dark field. (**b**) Trans-activation activity of ScDREB10 in yeast. Yeast cells yeast two-hybrid (Y2H) expressing the fusion proteins were cultured and adjusted to an OD600 of 2.0, then series diluted and dropped with 2 μL on nutritional selective medium without tryptophan (SD/−Trp), without tryptophan and histidine (SD/−Trp−His), and SD/−Trp−His with 5-Bromo-4-chloro-3-indolyl α-d-galactopyranoside (SD/−Trp−His+x-α-gal). Photos were taken after incubating at 30 °C for 2 to 4 days. Yeast cells expressing the empty vector pGBKT7-BD were used as negative control, and BD-GAL4 were used as positive control. SD: synthetic dropout.

The *35*:*ScDREB10-GFP* fusion gene and the *35S*:*GFP* control were transiently expressed in tobacco leaf epidermal cells to determine the localization of ScDREB10. As shown in Figure 2a, ScDREB10-GFP was distributed both in the nucleus and cytosol of tobacco leaf epidermal cells, as it is for free GFP, however, ScDREB10-GFP appears to be associated with the nuclear membrane (speckle-like patterns) rather than distributed throughout the whole nucleus as does free GFP (Figure 2a). To examine the transactivation activity of ScDREB10, the Y2H system was transformed with the fusion construct BD-ScDREB10, the negative control BD, and the positive control BD-GAL4, respectively. The yeast cells containing BD-*ScDREB10* or BD-GAL4 grew well in the SD/−TrpHis medium, whereas yeast cells containing negative control pGBKT7-BD did not grow (Figure 2b). Furthermore, in the presence of x-α-gal, the yeast cells harboring BD-*ScDREB10* and BD-GAL4 that grew well on the SD/−Trp−His medium turned blue (Figure 2b). These results confirmed that ScDREB10 has trans-activation activity in a yeast system.

### 3.3. Overexpression of ScDREB10 Increased the Osmotic and Salt Stress Tolerances in Arabidopsis at the Germination Stage

**Figure 3:**
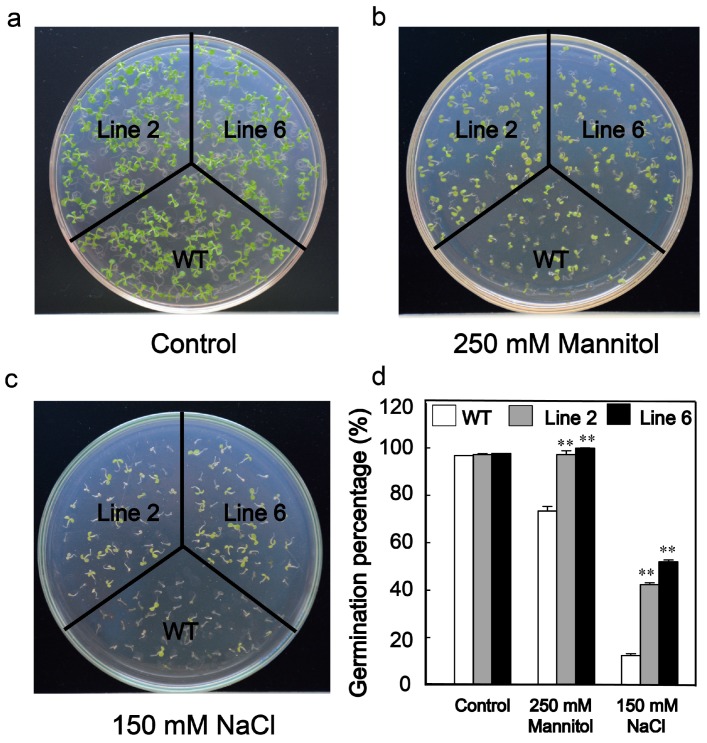
Determination of seed germination percentage under osmotic and salt stresses. Comparison of germination levels between wild type (WT) and *ScDREB10*-transformed seeds under normal (**a**), 250 mM mannitol (**b**), and 150 mM NaCl (**c**) treatment conditions. The *Arabidopsis* ecotype Col-0 was used as WT. (**d**) Germination percentage of WT and *ScDREB10*-transformed seeds under mannitol and NaCl treatment conditions 7 days after sowing. The germination percentage was calculated as the number of germinated seeds divided by the total number of seeds, represented as a percentage. Values are means ± SE of three replicates (*n* = 40–60 seeds). Asterisks indicate statistically significant differences from WT (** *P* < 0.01).

Transgenic *Arabidopsis* plants overexpressing *ScDREB10* were generated, and both PCR and RT-PCR analysis confirmed that the exogenous *ScDREB10* was successfully expressed in the transgenic lines (Figure S1). Two lines (line 2, line 6) of transgenic plants were selected for further functional evaluation studies. The germination percentage was calculated using means ± SE of three replicates (*n* = 40–60 seeds). The data were analyzed by LSD multiple comparison tests in the one-way analysis of variance. Germination percentage showed that there was no difference in seed germination under standard growth conditions (Figure 3). However, the germination percentage of the transgenic lines were 98 and 96%, respectively, compared to 73% for the WT control in the osmotic stress, and showed 42 and 52% compared to 12% for the WT control in the salt stress (** *P* < 0.01) (Figure 3). These results indicated that overexpression of *ScDREB10* gene is associated with increased osmotic and salt stress tolerances in *Arabidopsis* at the germination stage.

### 3.4. Overexpression of ScDREB10 Improved the Osmotic and Salt Stress Tolerances of Transgenic Arabidopsis at Seedling Stage

**Figure 4:**
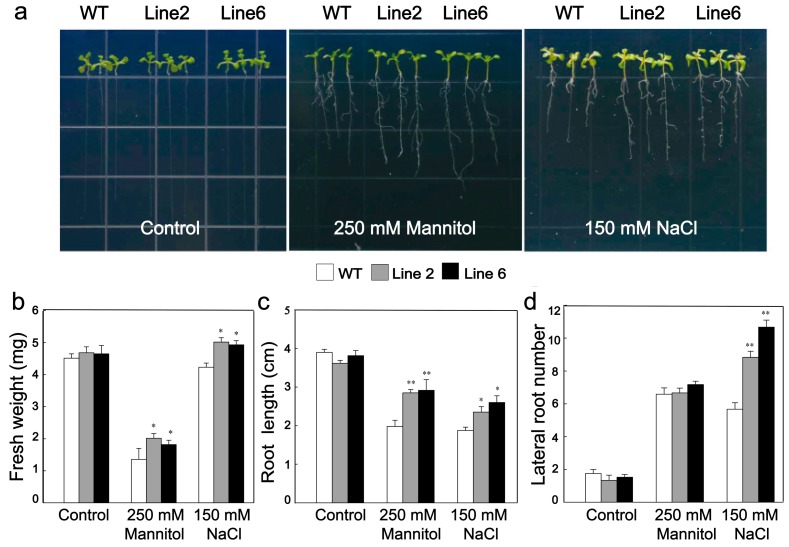
Growth of WT plants and two *ScDREB10* transgenic *Arabidopsis* lines under osmotic and salt stresses at seeding stage. One-week-old seedlings of WT and transgenic *Arabidopsis* cultured on Murashige and Skoog (MS) medium were transferred on the MS agar plates (control), MS agar plates supplied with 250 mM mannitol (osmotic stress) or 150 mM NaCl (salt stress), and maintained for 7 days. (**a**) Growth of WT and transgenic *Arabidopsis* plant on MS medium (control) and MS medium supplied with 250 mM mannitol and 150 mM NaCl for 7 days. (**b**) Comparison of fresh weight between WT and transgenic plants. (**c**) Comparison of root length between WT and transgenic plants. (**d**) Comparison of lateral root number between WT and transgenic plants. More than 20 plants of WT and each transgenic seedling lines were used in this experiment for further calculating the fresh weight, length root, and lateral root number. Values are means ± SE of at least twenty plants). Asterisks indicate statistically significant differences from WT (* *P* < 0.05, ** *P* < 0.01).

The growth of 7-day-old seedlings transgenic and WT plants was compared following exposure to osmotic stress (MS medium containing 250 mM mannitol) and salt (MS plates containing 150 mM NaCl). There were no differences in phenotypes, fresh weight, and root length between WT and transgenic plants under non-stress condition (Figure 4a,b). However, transgenic plants have significant higher fresh weight and longer root length than the WT plants after exposure to NaCl and mannitol treatments (Figure 4b,c). In addition, the lateral root numbers of transgenic plants were considerably increased compared with WT plants under the salt treatment (Figure 4d). These results indicated that overexpression of *ScDREB10* conferred enhanced tolerance to osmotic and salt stresses in transgenic plants.

### 3.5. ScDREB10 Improved ROS Scavenging Capability and Decreased the ROS Damage of Transgenic Arabidopsis under Osmotic and Salt Stresses

**Figure 5:**
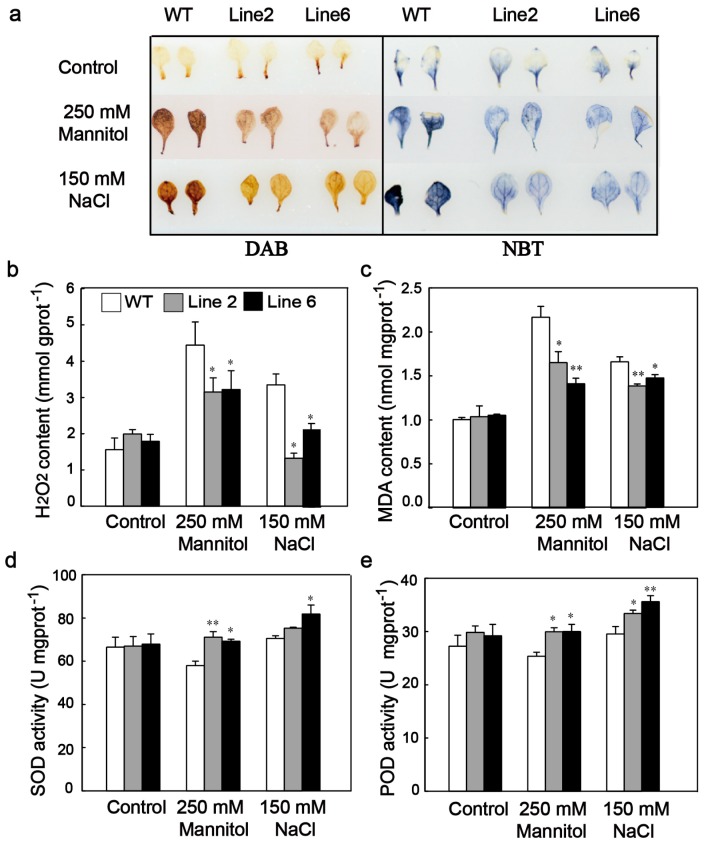
Comparison of reactive oxygen species (ROS) levels and antioxidant enzyme activities between WT and transgenic *Arabidopsis* under osmotic and salt stresses. One-week-old seedlings of WT and transgenic *Arabidopsis* cultured on MS medium were transferred on the MS agar plates (control), MS agar plates supplied with 250 mM mannitol (osmotic stress) or 150 mM NaCl (salt stress), and maintained for 7 days, then leaves were collected for histochemical staining and physiological indexes measurement. (**a**) Histochemical detection of hydrogen peroxide (diaminobenzidine (DAB) staining) and superoxide anion (nitrotetrazolium blue chloride (NBT) staining) accumulation. Measurement of H_2_O_2_ and malondialdehyde (MDA) contents (**b**,**c**). Measurement of superoxide dismutase (SOD), peroxidase (POD) activities (**d**,**e**) from 7-day-old seedlings. Values are means ± SE of three replicates. Asterisks indicate statistically significant differences from WT (* *P* < 0.05, ** *P* < 0.01).

Abiotic stresses can lead to oxidative damages due to the increased production of ROS. Therefore, it is important for plants to activate anti-oxidative systems coping with oxidative damage. DAB and NBT staining were used to determine the two main ROS species (H_2_O_2_ and superoxide anion, O_2_^−^) accumulation in transgenic *Arabidopsis* and WT plants under osmotic and salt stresses. There were no significant differences in H_2_O_2_ or O_2_^−^ levels between transgenic and WT plants under standard growth conditions (Figure 5a,b). However, the ROS accumulation level was lower in transgenic plants as compared with WT plants under both osmotic and salt stress conditions (Figure 5a). Consistently, measurement of H_2_O_2_ content also showed that transgenic *Arabidopsis* accumulated less H_2_O_2_ than WT under both osmotic and salt stresses (Figure 5b). MDA content was analyzed to investigate membrane lipid peroxidation levels. Likewise, the MDA content showed no difference between WT and transgenic plants under standard growth condition. However, the transgenic *Arabidopsis* accumulated less MDA than WT under the osmotic and salt stresses (Figure 5c). In addition, SOD and POD activities induced by mannitol and NaCl treatments were analyzed in transgenic and WT plants (Figure 5d,e). The results showed that the activities of SOD and POD were significantly higher in transgenic lines than WT plants under both osmotic and salt stresses. These results indicated that overexpression of *ScDREB10* can improve ROS scavenging ability, which may lead to lower ROS accumulation and limit cell damage in transgenic *Arabidopsis* compared with WT plants under osmotic and salt stress conditions.

### 3.6. Analysis of the Potential Downstream Genes Regulated by ScDREB10 in Response to Osmotic and Salt Stresses

**Figure 6:**
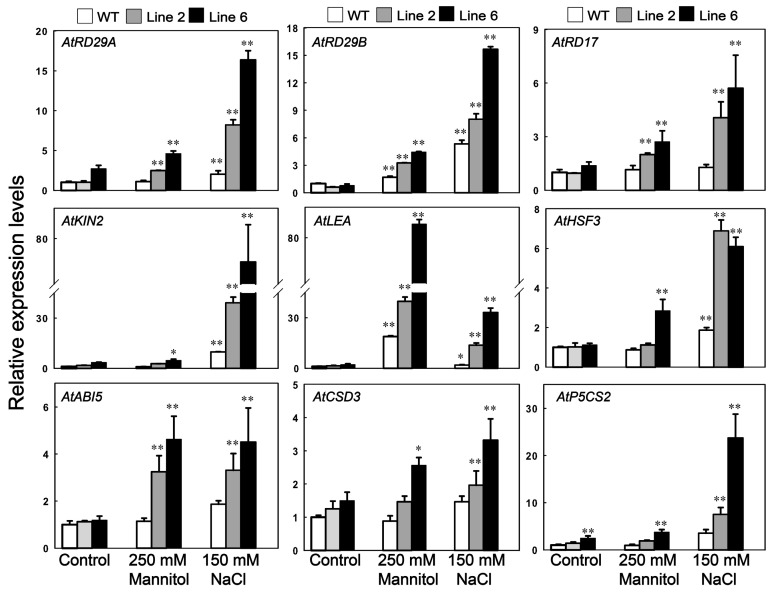
Gene expression level analysis of potential downstream genes of *ScDREB10* in response to osmotic and salt stresses. One-week-old seedlings of WT and transgenic *Arabidopsis* cultured on MS medium were transferred on the MS agar plates (control), MS agar plates supplied with 250 mM mannitol (osmotic stress) or 150 mM NaCl (salt stress), and maintained for 7 days. Abiotic stress-responsive genes were analyzed by RT-qPCR in WT and *ScDREB10* transgenic plants after osmotic and salt treatments. Total RNAs were extracted from leaves, and RT-qPCR analysis was performed. The 2^−ΔΔ*C*t^ method was used in RT-qPCR analysis. The *α-TUBULIN* (At1g50010) and *UBIQUITIN10* (At4g0532) genes of *Arabidopsis* were used as the reference genes. Values are means ± SE of three replicates. Asterisks indicate statistically significant differences from WT (* *P* < 0.05, ** *P* < 0.01).

To test if *ScDREB10* could regulate the classic stress-related downstream gene expression, RT-qPCR analysis was performed to examine the expression of nine abiotic stress-responsive genes in transgenic and WT plants under normal and stress conditions. Five genes responsive to dehydration including three RD (responsive to dehydration) genes (*AtRD29A*, *AtRD29B*, *AtRD17*), *AtLEA* (late embryogenesis abundant) and *AtABI5* (ABA insensitive 5); two genes responsive to low/high temperature including *AtKIN2* (cold-responsive 6.6) and *AtHSF3* (heat shock transcription factor 3); two genes associated with SOD and proline biosynthesis: *AtCSD3* (copper/zinc superoxide dismutase 3) and *AtP5CS2* (pyrroline-5-carboxylates synthetase). Under standard growth conditions, all these genes except *AtCSD3* and *AtP5CS2*, showed no notable differences in the expression between transgenic and WT plants (Figure 6), *P5CS2* has been confirmed to be associated with proline accumulation, and this transcript was significantly elevated in the *ScDREB10* transgenic plants under both normal and stress conditions compared with the WT plants (Figure 6). *AtCSD3* gene associated with the SOD was also significantly higher in transgenic plants compared to the WT plants. Relative expression of *AtRD29A*, *AtRD29B*, *AtRD17*, and *AtKIN2*, which contain DRE elements in their promoter regions, were significantly increased in the transgenic lines under stress condition compared with the WT. Likewise, other stress-responsive genes, *AtLEA*, *AtHSF3*, *AtABI5*, were also increased significantly under osmotic and salt treatments. These results showed that *ScDREB10* might play important roles in the responses to osmotic or salt stress condition by inducing various abiotic stress-responsive downstream genes.

## 4. Discussion

### 4.1. ScDREB10 is an A-5c Type of DREB Gene Which Can Improve Plant Osmotic and Salt Tolerance without Growth Penalty

Most studies on *DREB* genes have focused on the A-1 (*DREB1*) and A-2 (*DREB2*) groups since these gene products have been demonstrated to play dominant roles in plant abiotic stress response [9,11,36,37,38]. The study of A-5 group DREBs, which represent a large proportion of the DREB subfamily, is limited [19]. Although few of A-5 DREB proteins, such as RAP2.1 and MsDREB5, were reported to negatively regulate drought and cold stress response in *Arabidopsis* [39,40]. However, most of A-5 DREBs can improve at least one kind of plant stress tolerance. For example, *StDREB2* enhanced salt tolerance of transgenic potato [41], and soybean *GmDREB3* gene improved drought, salt and cold stress tolerance in transgenic *Arabidopsis* [42]. Recent functional studies on A-5 group members suggest that A-5 DREBs also play important roles in plant abiotic stress response [19,26,42,43]. Additionally, A-5 DREB can be further divided into three subgroups (A-5a, A-5b, and A-5c). All reports regarding the functions of A-5 type DREBs focused on the A-5a subgroup DREBs, such as RAP2.1 from *Arabidopsis* [39], GhDBP from cotton [44], and GmDREB2 from soybean [45]. No functional analysis has been done for A-5c DREB proteins until now. 

Based on the transcriptome data of the desert moss *S. caninervis*, ten stress-inducible A-5 types of *ScDREBs* were identified, and a sub-set showed strong stress tolerance in transgenic yeast [24,25]. Previously, we reported that *ScDREB8* belonged to A-5a type of *ScDREBs* and improved the salt stress tolerance of transgenic *Arabidopsis* [26]. In this study, we investigated the stress tolerance function of *ScDREB10* which belongs to the A-5c type of DREBs. Similar to *GmDREB2* from soybean and *HhDREB2* from *Halimodendron halodendronand* (A-5a DREBs; [45,46]), *ScDREB10* can significantly improve both salt and osmotic stress tolerance of transgenic *Arabidopsis*. In contrast to *ScDREB10*, *GmDREB3* (soybean) and *PpDBF1* (*Physcomitrella patens*) improve drought, salt, and cold tolerance [42,43]. Initial classification schemes for DREBs suggested that *DREB1* genes were primarily cold-induced while *DREB2* genes were induced in response to salt, drought, and heat. The functional division among different groups of DREB proteins (A1-A6 group) is less clear than originally supposed [15], and our results strengthen the idea that A-5 type DREBs play important roles in plant stress response(s).

Most of the reported *DREB* genes increased stress tolerance of overexpression transgenic plants, but are also associated with growth defect compared to wild-type plants, such as overexpression of *GmDREB2A* and *OsDREB1A* genes, which severely affected the growth of transgenic *Arabidopsis* [8,47]. It is suggested that this dwarf phenotype may be because of constituting expression of stress and grow related genes driven by the 35S promoter [15,48]. However, overexpression of a small number of *DREB* genes under the control of the 35S promoter produced no negative changes in phenotypes of transgenic plants, such as *AtDREB1A* and *CAP2* genes [49,50]. Similarly, in the present study, *ScDREB10* overexpression transgenic *Arabidopsis* did not cause any phenotype change compared with WT plants in normal growth condition, and *ScDREB10* overexpression *Arabidopsis* showed better stress tolerances with better growth performance (greater fresh weight) at the young seedling stage compared with WT plants under stress conditions. Shen et al. reported that the monocot gene transferred to dicots may not function as effectively as in the monocots [51], and Agarwal et al. deduced that the *DREB* gene from donor species may be less optimized for heterological expression in other plants, so the strong overexpression of *DREB* genes may be compensated to some degree [15]. The *ScDREB10* gene from moss plant shared very low sequence similarity with *DREB* genes in other plants (Figure 1, Table S3), so the dwarf growth was not found for transgenic ScDREB10 *Arabidopsis* which may be explained by being less optimized for heterological expression.

### 4.2. ScDREB10 Accumulation Enhances ROS Scavenging Ability and Upregulates the Expression of Many Stress-Related Genes under Stress Conditions

Various abiotic stresses can lead to increased production ROS and other radicals which can cause oxidative damage to proteins, DNA, and lipids [52,53]. Therefore, it is important for plants to activate anti-oxidative systems coping with oxidative damage [54]. Research shows that antioxidant systems have an important role in plant response to various abiotic stresses [55,56,57], and antioxidant enzymes, such as SOD and POD, are important ROS-scavenging enzymes that play critical roles in modulating ROS levels induced by abiotic stress [58]. In this work, ROS accumulation was first analyzed by DAB and NBT histochemical staining. Our result showed that *ScDREB10* transgenic *Arabidopsis* showed lower ROS damage during osmotic and salt stresses compared with WT plants. Consistent with this result, lower H_2_O_2_ levels were detected in transgenic plants. MDA contents also showed that O_2_^−^, H_2_O_2_ and membrane lipid peroxidation levels were strongly decreased in transgenic plants. Subsequently, we further compared the activities of the main ROS scavenging reagents SOD/POD in transgenic and WT plants. Our results showed that under osmotic and salt stress conditions, the activities of SOD and POD were greatly improved in transgenic plants relative to that in WT plants. In general, these results suggest that *ScDREB10* conferred stress tolerance through enhancing the antioxidant enzyme activities of transgenic *Arabidopsis*, resulting in the decrease of ROS level to protect the plant from oxidative damage under osmotic and salt stress conditions.

Over-expression of *DREB* genes led to the accumulation of stress-inducible down-stream genes, such as LEA proteins and heat-shock-related proteins, thus, providing enhanced stress tolerance to plants [16]. As discussed above, ScDREB10 transgenic *Arabidopsis* enhances ROS scavenging ability to improve stress tolerance. Consistent with this result, *Arabidopsis* Cu/Zn superoxide dismutase gene *AtCSD3* was highly induced by mannitol and salt treatments, suggesting that *ScDREB10* might induce the expression of *AtCSD3* gene to increase SOD activity during osmotic and salt stresses. Except for *SOD* gene, sets of classic stress responsive genes, such as *RD29*, *RD17*, *LEA*, and *KIN2* genes were strongly upregulated by *ScDREB10* overexpression *Arabidopsis* under stress conditions (Figure 6), consistent with most studies in which overexpression of a DREB transcription activator may activate a series of down-stream genes that function in adapting to abiotic stresses [7,9,26,42]. However, unlike most of *DREB* genes, ScDREB10 only upregulated these stress-related genes under stress conditions rather than normal conditions with the exception of P5CS2 gene (Figure 6). This stress-inducible up-regulation of target genes may be one of the reasons that the dwarf growth was not found for transgenic ScDREB10 *Arabidopsis* under normal growth condition. Another explanation is that, as discussed above, since moss gene *ScDREB10* shared very low degrees sequence identity with other *DREBs*, the DNA/protein binding ability, strength, and specificity may be different so the numbers and types of target genes induced by heterologous overexpression of *ScDREB10* gene may be different compared with other DREBs.

### 4.3. ScDREB10 may Exert a Regulatory Function near the Nuclear Membrane

The subcellular localization is an important functional characteristic of proteins and plays an important role in the prediction of protein function [59]. To properly execute their biological functions, proteins must be targeted to the correct subcellular organelles. Considering their function in transcriptional regulation, transcriptional factors (TF) are postulated to be localized to the nucleus, and, in fact, many important TFs are demonstrated to be localized within the nucleus [60,61]. However, some TFs also shown other localizations. For example, AaORA (octadecanoid responsive AP2-domain protein) was reported to localize both to the nucleus and cytosol [62], and NTM2 protein was localized at the plasma membranes [63]. Additionally, some TFs showed sub-nuclear localization, such as AtERF069, that localized within the nucleoplasm and were excluded from nucleolus (nuclear speckle ring-like pattern) [64]. Among of DREB TFs, the majority was reported to be localized in the nucleus [7,8,13,39,40]. Previously, we reported that ScDREB8 identified from *S. caninervis* resided both in the nucleus and cytosol. In this study, similar to ScDREB8, ScDREB10 protein also showed a nucleocytosolic localization. However, unlike ScDREB8, the ScDREB10 protein appears to have a strong association with the nuclear membrane. ScDREB10 may exert a regulatory function near the nuclear membrane. Factors, such as relocalization upon stimulus or protein interaction [64], can influence protein localization in vivo. To understand this specific nuclear membrane expression pattern of ScDREB10, we should take these factors into account and further test the location of ScDREB10 in future.

## Supplementary Materials

The following are available online at http://www.mdpi.com/2073-4425/10/2/146/s1. Figure S1: Molecular identification of the *ScDREB10* transgenic *Arabidopsis* lines, Table S1: List of primers used for vector construction, Table S2: List of primers used for real-time PCR, Table S3: Sequence similarity analysis of 22 DREB proteins and estimates of evolutionary divergence between sequences.
