# Supplementary figures and images for: ScDREB10, an A-5c type of DREB Gene of the Desert Moss Syntrichia caninervis, Confers Osmotic and Salt Tolerances to Arabidopsis
Source: Genes (Basel). 2019 Feb 14;10(2):146. doi: 10.3390/genes10020146 (PMC6409532; doi:10.3390/genes10020146)

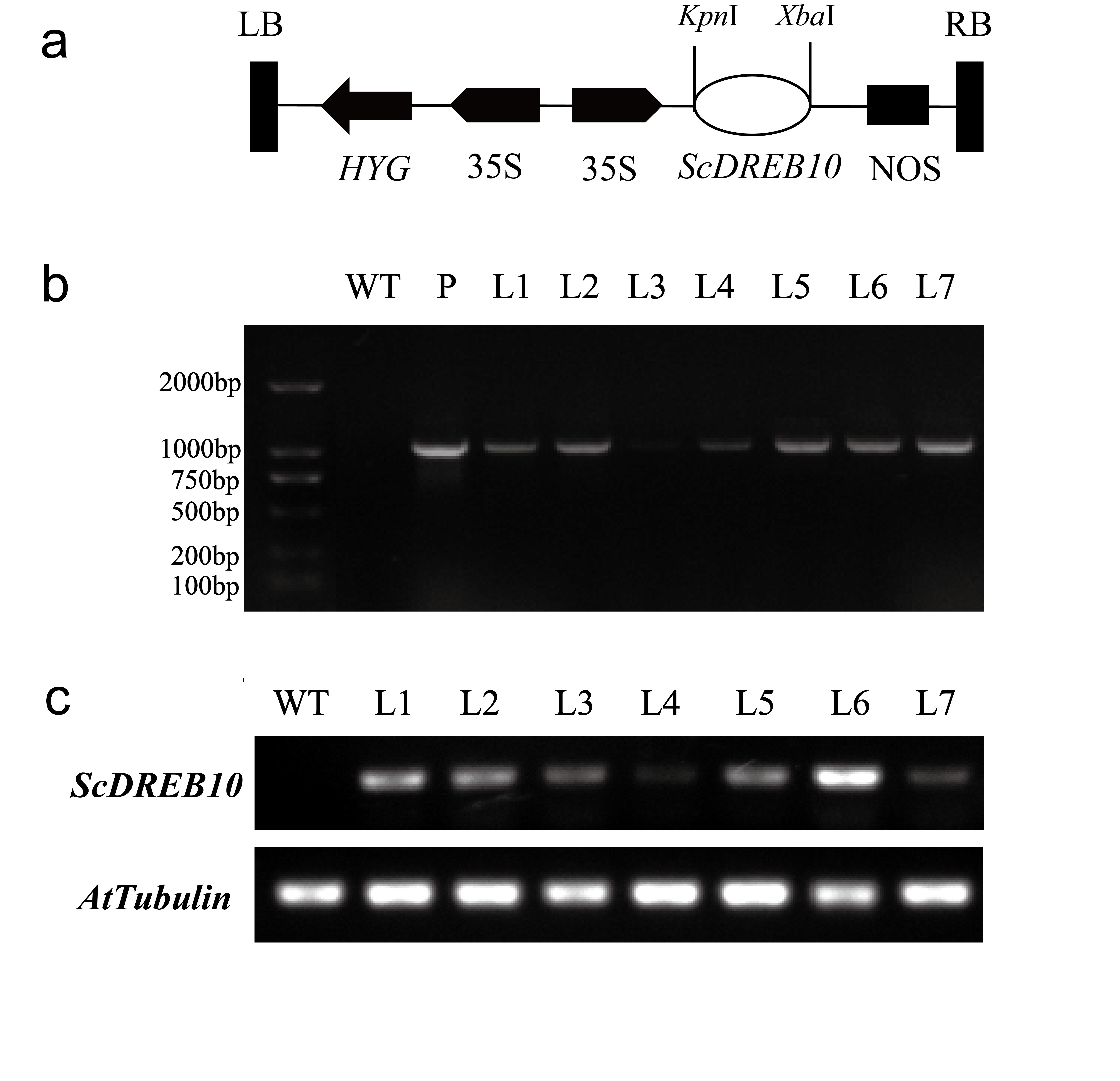

Supplement: Supplementary file 1 [file genes-10-00146-s001.zip › supplementary files/Figure S1.jpg]
