# Supplementary material for: ScDREB10, an A-5c type of DREB Gene of the Desert Moss Syntrichia caninervis, Confers Osmotic and Salt Tolerances to Arabidopsis
Source: Genes (Basel). 2019 Feb 14;10(2):146. doi: 10.3390/genes10020146 (PMC6409532; doi:10.3390/genes10020146)
Supplement: Supplementary file 1 [file genes-10-00146-s001.zip › supplementary files/Table S1,S2.doc]

Supporting Information

Table S1 List of primers used for vector construction

The vector sequences were underlined

| Primer | Sequence (5’ to 3’) |
| --- | --- |
| pBI121-GFP-F | TTTCATTTGGAGAGAACACG |
| pBI121-GFP-R  pBI121(in-fusion)-*ScDREB10*-F  pBI121(in-fusion)-*ScDREB10*-R | CGACCAGGATGGGCACCAC  TCTAGACTGGTACCCATGGCCGGCGAAGAAGCT  CTAGTCAGTCGACCCTTCCGGATGTCTGCGGAT |
| pGBKT7-F | GAATTCCCGGGGATCCATGCCTGGGATGGCACCTTC |
| pGBKT7-R  pGBKT7(in-fusion)-*ScDREB10*-F pGBKT7(in-fusion)-*ScDREB10*-R | T AGTTATGCGGCCGCTGCAGTCAAAACCCTCGGTGGCG  GAATTCCCGGGGATCCATGGCCGGCGAAGAAGCT  TAGTTATGCGGCCGCTGCAGTCACCGGATGTCTGCGGATG |

Table S2 List of primers for real time PCR

| Gene name | AGI No. | Primers (5’ to 3’) |
| --- | --- | --- |
| *AtTubulin* | At1g50010 | F: GATGTACCGTGGTGATGTC |
|  |  | R: GAGCCTCTGAAAATTCTCC |
| *AtUBQ10* | At4G05320 | F: CACACTCCACTTGGTCTTGCGT |
|  |  | R: TGGTCTTTCCGGTGAGAGTCTTCA |
| *AtRD29A* | At5g52310 | F:TGGATCTGAAGAACGAATCTGATATC |
|  |  | R:GGTCTTCCCTTCGCCAGAA |
| *AtRD29B* | At5g52300 | F: AAACGGCTACCACATCCAAG |
|  |  | R: CCTCCAATGGATCCTCGTTA |
| *AtRD17* | At1g20440 | F: ACGTCCACGCCGTTGGT |
|  |  | R: CTCCGGATGTTCCACTGGAA |
| *AtKIN2* | [At5g15970](http://www.arabidopsis.org/servlets/TairObject?type=locus&name=AT5G15970) | F: GTCAGAGACCAACAAGAATGCC |
|  |  | R: TGACTCGGATCGCTACTTGTTC |
| *AtLEA* | At1g52690 | F: GCAATCAAGAACAAGGCACA |
|  |  | R: TCAGTGCGAAGCCCTAAAGT |
| *AtHSF3* | At5g03720 | F: TTCGCTAACGAGGCTTTCC |
|  |  | R: CCTCAGTAGGTGACCCTT |
| *ABI5* | At2g36270 | F:AAATTGCCGAAATCGAACAG |
|  |  | R:TTTTCCCTCGTTCTGTCTCC |
| *AtCSD3* | At5g18100 | F: AACGAGGAAGAGCGTCATG |
|  |  | R: GCGTTTCCAGTTGATTTG |
| *AtP5CS2* | At1g55610 | F: ATGATCTTATTTATGTTCTGC |
|  |  | R: CACTATCTTCCGTCACTAT |
|  |  |  |
|  |  |  |
|  |  |  |
|  |  |  |
|  |  |  |
|  |  |  |
|  |  |  |
